# Supplementary material for: Current Prevalence of Oral Helicobacter pylori among Japanese Adults Determined Using a Nested Polymerase Chain Reaction Assay
Source: Pathogens. 2020 Dec 24;10(1):10. doi: 10.3390/pathogens10010010 (PMC7824695; doi:10.3390/pathogens10010010)
Supplement: Supplementary file 1 [file pathogens-10-00010-s001.pdf]

```
NCTC 11637     GGCAAATCATAAGTCCGCAGAAAAGCGAATCAGACAGACCATTAAAAGAACCGAACGCAACAGGTTCTATAAAACTAAAAT
Oral sample    GGCAAATCATAAGTCCGCAGAAAAGCGAATCAGACAGACCATTAAAAGAACCGAACGCAACAGGTTCTATAAAACTAAAAT
               *********************************************************************************

NCTC 11637     TAAAAATATCGTTAAAGCCGTGCGTGAAGCGGTCGCTGTCAATGATGTAACAAAAGCTCAAGAGCGTTTGAAAATCGCTAA
Oral sample    TAAAAATATCGTTAAAGCCGTGCGTGAAGCGGTCGCTGTCAATGATGTAACAAAAGCTCAAGAGCGTTTGAAAATCGCTAA
               *********************************************************************************

NCTC 11637     TAAAGAGTTGCATAAATTTGTCAGCAAAGGGATTTTAAAGAAAAACACCGCTTCTAGGAAAGTCTCA
Oral sample    TAAAGAGTTGCATAAATTTGTCAGCAAAGGGATTTTAAAGAAAAACACCGCTTCTAGGAAAGTCTCA
               *******************************************************************
```

Figure S1: Representative nucleotide sequences of 228-bp fragments from 43 oral samples judged positive by nested PCR. Dashes indicate nucleotide identity compared with the standard sequence from *H. pylori* IID3023.

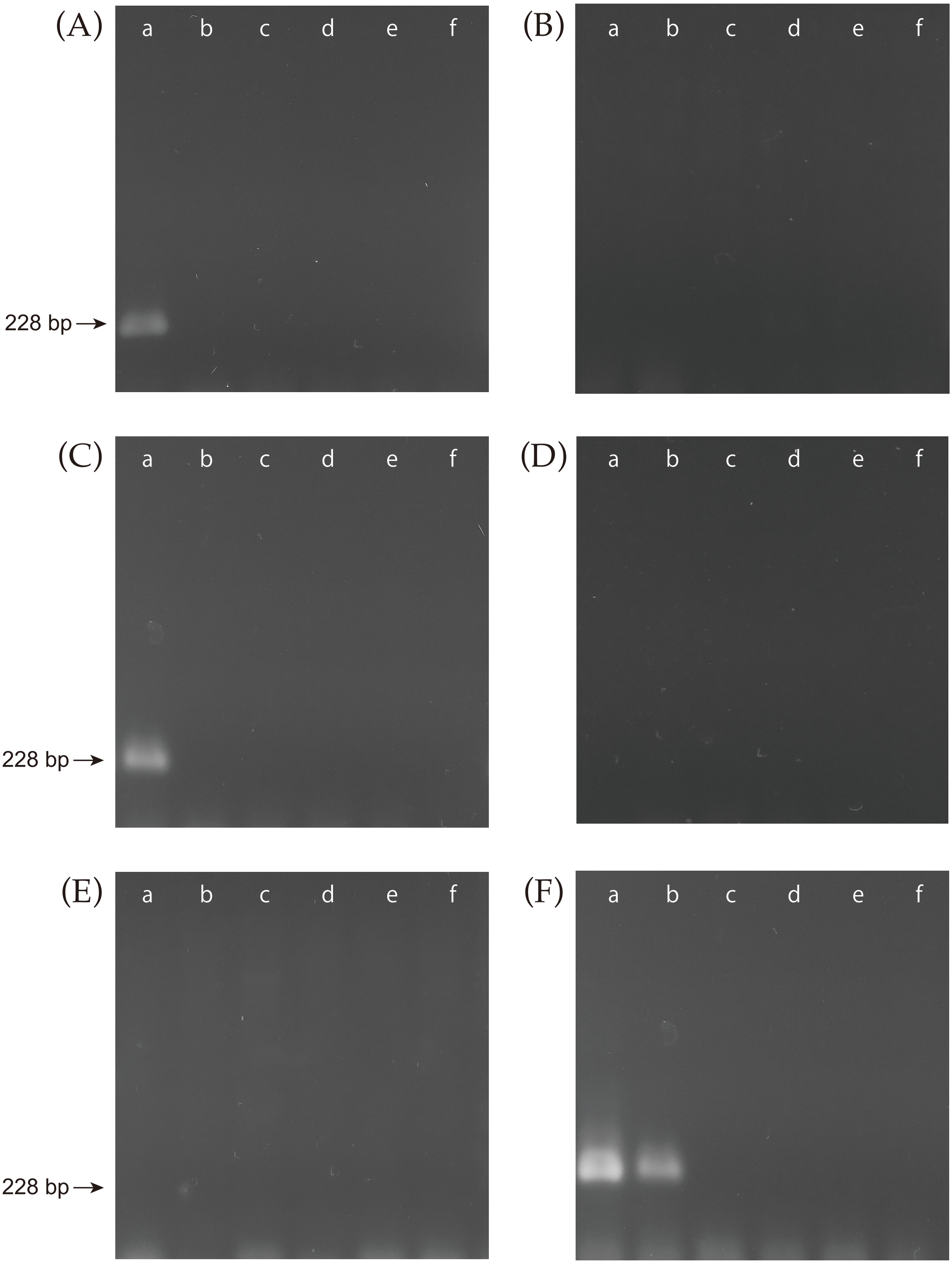

Figure S2. Agarose gel electrophoresis image of oral samples collected from a participant who received successful eradication therapy. One participant who received successful eradication therapy provided supragingival biofilm every 2 month, and the presence of oral *H. pylori* was examined using nested PCR. A: March 27, 2019, B: September 30, 2019, C: October 30, 2019, D: December 3, 2019, E: February 14, 2020, F: July 22,2020; a, upper incisors; b, lower incisors; c, upper right molars; d, lower left molars; e, tongue; f, saliva.
